# Evolution of Resistance to Aurora Kinase B Inhibitors in Leukaemia Cells

**DOI:** 10.1371/journal.pone.0030734

**Published:** 2012-02-16

**Authors:** Timothy W. Failes, Gorjana Mitic, Heba Abdel-Halim, Sela T. Po'uha, Marjorie Liu, David E. Hibbs, Maria Kavallaris

**Affiliations:** 1 Childrens Cancer Institute Australia, Lowy Cancer Research Centregh, University of New South Wales, Randwick, Australia; 2 Faculty of Pharmacy, University of Sydney, Sydney, Australia; 3 Australian Centre for Nanomedicine, University of New South Wales, Sydney, Australia; Vanderbilt University Medical Center, United States of America

## Abstract

Aurora kinase inhibitors are new mitosis-targeting drugs currently in clinical trials for the treatment of haematological and solid malignancies. However, knowledge of the molecular factors that influence sensitivity and resistance remains limited. Herein, we developed and characterised an in vitro leukaemia model of resistance to the Aurora B inhibitor ZM447439. Human T-cell acute lymphoblastic leukaemia cells, CCRF-CEM, were selected for resistance in 4 µM ZM447439. CEM/AKB4 cells showed no cross-resistance to tubulin-targeted and DNA-damaging agents, but were hypersensitive to an Aurora kinase A inhibitor. Sequencing revealed a mutation in the Aurora B kinase domain corresponding to a G160E amino acid substitution. Molecular modelling of drug binding in Aurora B containing this mutation suggested that resistance is mediated by the glutamate substitution preventing formation of an active drug-binding motif. Progression of resistance in the more highly selected CEM/AKB8 and CEM/AKB16 cells, derived sequentially from CEM/AKB4 in 8 and 16 µM ZM447439 respectively, was mediated by additional defects. These defects were independent of Aurora B and multi-drug resistance pathways and are associated with reduced apoptosis mostly likely due to reduced inhibition of the catalytic activity of aurora kinase B in the presence of drug. Our findings are important in the context of the use of these new targeted agents in treatment regimes against leukaemia and suggest resistance to therapy may arise through multiple independent mechanisms.

## Introduction

Mitotic kinases play crucial roles in regulation of cell division, yet aberrations in their expression and function are known to be involved in cancer initiation and progression. Targeting these kinases has proven in recent years to be an exciting avenue for alternative cancer therapies [1]. The Aurora kinases have emerged as particularly promising targets due their roles in regulating multiple signalling pathways crucial for accurate cell division. Localization and function of each subtype – Aurora A, B and C, has been studied and reviewed extensively in the recent literature [2], [3]. The association and implication of the Aurora kinases in cancer stems from early studies that revealed aberrant expression of both Aurora A and B in many solid and hematological malignancies. This association of Aurora kinase overexpression with a malignant phenotype has been functionally validated [4], [5], [6], [7], [8]. Deregulation of the Aurora kinases disrupts mitotic processes crucial for accurate cell division leading to chromosomal instability and aneuploidy [9], [10] however a complete understanding of their role in tumourigenesis remains elusive. Reports of the role and function of Aurora A and B in leukaemia have been largely limited to expression studies in cell lines and small cohort clinical studies. Increased expression of Aurora A has been reported in many leukaemias, while the expression of Aurora B has shown no clear trend [11], [12], [13]. Despite this, both Aurora A and B have been exploited as potential targets for therapeutic intervention.

The promise of the Aurora kinases as anticancer targets has been such that small molecule inhibition as drug therapy is a rapidly developing area of research [2], [14]. Early successful candidates in preclinical testing were pan-Aurora inhibitors such as VX-680 [15], however it was shown that the dominant phenotype arising from these agents was that of Aurora B inhibition [16]. Aurora B specific inhibitors such as AZD1152 [17] have since shown increasing promise and have reached early stage clinical trials against both solid and haematological malignancies. The earliest documented Aurora B inhibitor ZM447439 has also been well characterised as a probe of the cellular biology of Aurora B [18]. Cellular phenotypes of these agents such as inhibition of histone H3 phosphorylation, cytokinesis failure, and polyploidisation are consistent with inhibition of Aurora B.

As yet, however, the specific factors that will influence sensitivity and resistance to Aurora kinase inhibitors have not been adequately addressed. A major drawback of molecularly targeted agents is the likelihood of acquired clinical resistance. Early success of the BCR-ABL kinase targeting drug Imatinib in the treatment of chronic myelogenous leukaemia was followed by the rapid emergence of clinical resistance. Resistance was discovered to be mediated by point mutations in the kinase domain preventing drug binding but maintaining catalytic activity [19]. Identification of these resistance conferring mutations has led to the design of later-generation inhibitors that circumvent these changes and allowed successful treatment of Imatinib resistant patients [20]. Experience with other agents targeting a single kinase, such as for inhibitors of EGFR, FLT3, KIT and PDGFR kinases, shows resistance mediated by kinase domain mutations is a recurring theme.

It appears that resistance mediated by kinase domain mutations is also a distinct possibility for Aurora kinase inhibitors. A recent in vitro study reported four point mutations in colorectal cell lines selected for resistance to ZM447439, with functional studies showing that each mutation independently conferred a resistant phenotype [21]. These reported mutations in a colorectal cancer cell line may be just a subset of possible changes and it is not clear whether other point mutations would appear in other tumour types. Moreover, while clinical resistance can clearly be mediated through kinase mutations, the emergence of other novel resistance pathways in a clinical setting may be possible. Engagement of alternative survival pathways and the recently described “retreatment response” [22] upon multiple drug exposures are examples of non-mutational mechanisms in targeted drug resistance. The interplay of these independent resistance pathways and their relative contribution to a resistant phenotype is still unclear for most anticancer agents, particularly in a clinical context. An understanding of these networks is crucial in designing optimal treatment approaches for targeted therapies, such as Aurora B inhibitors.

In this study we report the development of a leukaemia resistance model and the characterisation of resistance mechanisms associated with the Aurora B inhibitor ZM447439. We also investigated the evolution of the resistance phenotype and show that multiple mechanisms of resistance emerge with increasing drug resistance levels.

## Materials and Methods

### Cell culture and selection of resistant cells

CCRF-CEM cells (a human T-cell acute lymphoblastic leukemia cell line [23]) were maintained as suspension cultures in RPMI-1640 (Gibco, Invitrogen, Mount Waverly, Victoria, Australia) medium containing 10% fetal calf serum (FCS; Gibco, Invitrogen). Resistant diploid CCRF-CEM cells were selected by four sequential treatments of 4 µM ZM447439 (Tocris, Bristol, UK) for 72 hr until cells were able to proliferate through treatment. After each treatment the population of viable cells was separated and recovered from dead cells by a published procedure [24]. The resulting resistant cell line designated CEM/AKB4 has since been maintained in drug free media. To generate sublines with higher levels of resistance, CEM/AKB4 cells were selected for growth in 8 µM and designated CEM/AKB8, and 16 µM ZM447439, designated CEM/AKB16. All cells used in this study were mycoplasma free.

### Growth inhibition assays

Growth inhibition assays were performed as previously described [25]. Briefly, cells were seeded at 15,000 cells/well in 96-well plates in the presence or absence of the indicated drug concentrations. Cytotoxic drugs were obtained as follows: AZD1152, MLN8237 (Selleck Chemicals, San Diego, CA) vincristine (Sigma); vinblastine (David Bull Laboratories, Lidcombe, NSW, Australia); doxorubicin (Pharmacia, Rydalmere, NSW, Australia); epothilone B and paclitaxel (Calbiochem, San Diego, CA); ENMD2076 (Entremed, Ontario, Canada). After 72 hr incubation, metabolic activity was detected by addition of Alamar blue and spectrophotometric analysis. Cell numbers were determined and expressed as a percentage of control, untreated cells. Determination of IC_50_ values and statistical analysis was performed as described previously [26].

### Cell cycle analysis by flow cytometry

Distribution of DNA content in CEM and CEM/AKB4 cells was determined by flow cytometry as previously described [27]. Briefly, cells were harvested, washed with PBS, and then stained for 15 min at 37°C with a solution containing 0.4% Triton X-100 (Sigma-Aldrich), 50 µg/mL of propidium iodide (Sigma-Aldrich), and 2 µg/mL of DNase-free RNase (Roche). The cells were then analyzed for cell cycle perturbation using a FACSCalibur (Becton Dickinson) flow cytometer. The CellQuest program was used to quantitate the distribution of cells in each cell cycle phase: sub-G_1_ (cell death), G_1_, S, and G_2_-M.

### Real-time PCR analysis

Total RNA was extracted using RNeasy Mini kits (Qiagen) according to the manufacturer's instructions and was used to prepare complementary DNA (cDNA) as previously described. The cDNAs were used to quantify gene expression for AurkB and MDR1 by real-time PCR using Taqman Gene Expression assays (Applied Biosystems) containing 6-carboxyfluorescein (FAM) labelled probes. PCR reactions were performed using the ABI Prism 7500 sequence detection system (Applied Biosystems) with a 25 µL reaction mixture containing 2 µL of cDNA template, 12.5 µL TaqMan Gene Expression PCR Master Mix (Applied Biosystems) and 1.25 µL Taqman assay. Cycling conditions were as follows: 50°C for 2 min, 95°C for 10 min, 40 cycles of 95°C for 15 s and 60°C for 1 min. Gene expression was normalised to the cyclophilin-A gene (PPIA) employed in multiplex using a TaqMan Endogenous Control assay (Applied Biosystems).

### Western blot analysis

Total cell lysates were separated by SDS-PAGE and electrotransferred to nitrocellulose membrane using standard methods. Primary antibodies used were rabbit monoclonal anti-Aurora kinase B ([EP1009Y], Abcam), rabbit monoclonal anti-phospho Histone H3(Ser10) ([D2C8], Cell Signaling), rabbit anti-cleaved PARP (Asp214) (Cell Signaling) and mouse monoclonal anti-GAPDH (glyceraldehyde 3-phosphate dehydrogenase) ([6c5], Abcam). Detection was performed using HRP-conjugated goat-anti-rabbit (Pierce) and sheep anti-mouse (Amersham) secondary antibodies. Bands were detected by the ECL Plus Western Blotting Detection reagent (GE Healthcare) and visualised and imaged on a Typhoon 9410 laser scanner (GE Healthcare). Relative expression is given as the ratio of the test band's densitometric volume to that of the respective GAPDH band.

### Immunofluorescence staining

Briefly, cells were plated in glass chamber slides and allowed to reach 70% confluence. Immunofluorescence staining was then done as described previously [28]. For dual staining, cells were first stained with an Aurora B antibody (Abcam) followed by Alexa-488 anti-mouse fluorescent-tagged antibody (GE Healthcare). This was then followed by staining with α-tubulin and Alexa-555 anti-mouse fluorescent-tagged antibody (GE Healthcare). Slides were mounted on a coverslip using DAPI II Counterstain (Vysis, Inc.). Immunofluorescence microscopy was done using a Zeiss Axioplan 2 Microscope (Zeiss), and images were captured using a Sensicam Charged Coupled Device camera (PCO Imaging) and the Image-Pro Plus 4.1 software (Media Cybernetics, L.P.).

### Mitotic Index

The parental CCRF-CEM and CEM/AKB resistant cells were either untreated or treated with 4 µM of Aurora B kinase inhibitor (ZM447439) for 24 hours and 5×10^4^ cells were cytospun onto glass slides. Mitotic index was determined as previously described [29]. At least 1000 interphase and mitotic cells were counted per condition from at least three independent experiments. Mitotic index was calculated by dividing the total number of mitotic cells by the total number of interphase and mitotic cells counted.

### Sequencing of AurkB gene

Gene sequencing was performed on cDNA from CEM and CEM/AKB4 cells as prepared above. Gene specific PCR primers were used to amplify the full length of the AurkB (accession no. NM_004217.2) coding region by using three overlapping primer sets. Sequences of the overlapping primer sets are as follows.

AKB -13F 5′TTTCTCTCTAAGGATGGCCC

AKB 328R 5′TGAAGAGGACCTTGAGCGCC

AKB 243F 5′TCCTCTGGGCAAAGGCAAG

AKB 638R 5′TCTCCCTTGAGCCCTAAGAG

AKB 508F 5′TGCACATTTGACGAGCAGCG

AKB 3′UTR 41R 5′AGACATACAAACACACGCACC

Amplification reactions had the following components: 1× PCR Gold Buffer (Applied Biosystems), 0.2 mM deoxynucleoside triphosphates (dNTPs), 250 ng of each primer, 1.5 mM MgCl_2_ solution (Applied Biosystems) and 2 U AmpliTaq Gold Polymerase (Applied Biosystems) in total volume of 50 µl. PCR cycle conditions were: 94°C 5 min, 35 cycles of 94°C 45 s, 57°C 45 s, 72°C 45 s followed by 72°C for 7 min. Amplified PCR products were resolved on a 1% agarose gel stained with crystal violet. The desired band was excised, DNA purified by using the QIAquick gel extraction kit (Qiagen) and sequenced with BigDye terminators. Sequence analyses were performed at the Sydney University Prince Alfred Molecular Analysis Centre (SUPAMAC).

### Apoptosis assays

Cellular apoptosis was determined by measurement of cleaved PARP (cPARP). Briefly, CEM, CEM/AKB4 and CEM/AKB16 cells were treated with varying concentrations of ZM447439 for 24 h. Following treatment, cells were harvested and levels of cPARP determined by western blotting. Additionally, induction of apoptosis was determined by measurement of Annexin V-FITC (Becton Dickinson) using flow cytometry as described previously [27].

### Molecular modelling and docking

Docking was performed with Glide 5.0 [30] from Schrödinger®. Initially the Aurora B (xenopus laevis) crystal structures co-crystallised with ZM447439, hesperadin, and an aminothiazole inhibitor (pdb codes: 2VRX, 2BFY and 2VGP respectively) were individually imported into the Maestro 8.5 [31] graphical user interface; protein preparation and refinement was employed on all structures. The glycine 176 (G176) residues of Aurora B in the above structures were mutated to glutamate (E176) to generate the mutant structures and these structures were prepared and refined as before. The Protein preparation module allows the refinement of the protein crystal structure by deleting crystal water molecules, adding hydrogens, restoring bond orders and correcting any steric clashes among different amino acid residues. To use these structures for ligand docking the shape and properties of the receptor should be represented on a grid; the receptor grid generation module in Glide 5.0 was used to generate four different grids for each of the crystal structures and their corresponding mutants. The binding site to be used for docking was determined as a centroid of the crystal structure ligand position. The Coulomb-van der Waals (vdW) radii of the receptor residues were set as 1.

The docking process followed by flexibly docking each ligand into the corresponding wild-type and mutant protein structures, the extra precision (XP) function was used in all the docking runs and the vdW scaling of 1.0 was used for the ligands vdW radii. Ligands were built using the Maestro 8.5 [31] graphical user interface and were minimized with the MacroModel 9.6 [32] module using the OPLS_2005 force field [33].

### Statistical analysis

Statistical analysis was performed using the GraphPad Prism program. Results were expressed as means of at least three independent experiments ± SEM. A two-tailed Student's *t* test was used to determine the statistical differences between various experimental and control groups, with *P*<0.05 considered statistically significant.

## Results

### Selection of ZM447439 resistant leukaemia cells

**Figure 1:**
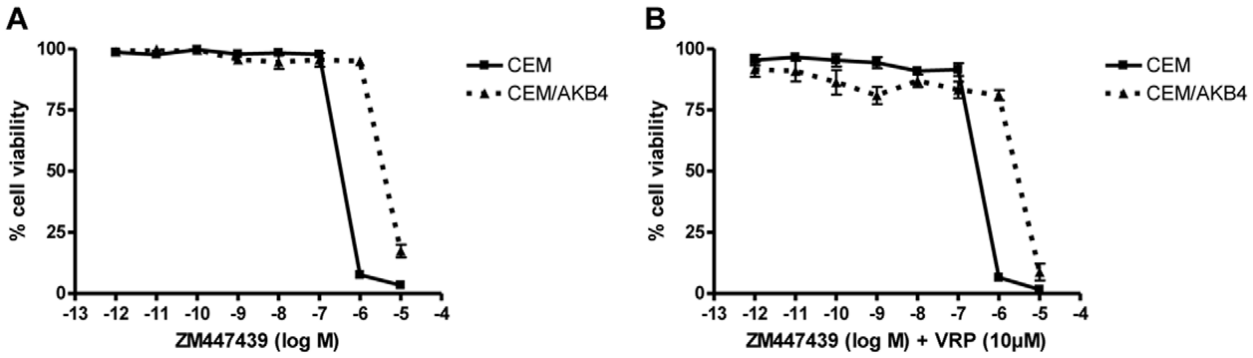
Resistance levels of CEM/AKB4. (A) Plot of cell viability against concentration of ZM447439 for both CEM/AKB4 and parental CEM cells as determined by cytotoxicity assay. (B) Results of the same experiment performed in the presence of the P-glycoprotein inhibitor verapamil. Points are the means, and bars are the SEM of at least three independent experiments.

Prior to developing Aurora B inhibitor resistant leukaemia cells cytotoxicity assays on CCRF-CEM T-cell leukemia cells were performed using ZM447439 (ZM). The IC_90_ for ZM against CCRF-CEM cells was 4 µM. Selection of a ZM-resistant CEM subline was achieved by sequential 72 hr treatments of CEM cells with 4 µM ZM followed by recovery and expansion of the surviving population. Resistance was defined as cells being able to proliferate in the presence of the IC_90_ drug concentration. Four 72 hr treatments of CEM cells with 4 µM ZM yielded a resistant population designated CEM/AKB4. To determine the levels of resistance of CEM/AKB4 cells to ZM, cytotoxicity assays were performed. The activity of the drug was approximately an order of magnitude lower in CEM/AKB4 cells relative to CEM cells (Fig. 1). The relative resistance of CEM/AKB4 was 13.2 fold when compared to parental CEM cells (Table 1).

**Table 1:** Relative resistance of CEM/AKB4 cells to cytotoxic agents compared to parental CCRF-CEM cells.

Drug	Cell line	IC_50_	Relative Resistance^a^	*P*
ZM447439	CEM	3.40±0.37×10^−7^	-	
	CEM/AKB4	4.55±0.29×10^−6^	13.4	<0.0001
AZD1152	CEM	3.49±0.36×10^−8^	-	
	AKB4	2.47±0.27×10^−7^	7.08	0.0015
ENMD2076	CEM	2.67±0.46×10^−7^	-	
	CEM/AKB4	1.37±0.42×10^−7^	0.513	0.1055
MLN8237	CEM	8.84±0.46×10^−8^	-	
	CEM/AKB4	5.74±0.27×10^−8^	0.65	0.003
Vincristine	CEM	2.07±0.29×10^−9^	-	
	CEM/AKB4	1.08±0.28×10^−9^	0.522	0.0686
Vinblastine	CEM	3.13±0.07×10^−9^	-	
	CEM/AKB4	3.23±0.03×10^−9^	1.03	0.2508
Paclitaxel	CEM	2.18±0.69×10^−8^	-	
	CEM/AKB4	8.8±5.6×10^−9^	0.404	0.2184
Epothilone B	CEM	2.27±0.12×10^−9^	-	
	CEM/AKB4	2.73±0.26×10^−9^	1.20	0.1790
Doxorubicin	CEM	5.39±1.54×10^−8^	-	
	CEM/AKB4	3.03±0.37×10^−8^	0.562	0.2120

a Determined by dividing the IC_50_ for the resistant (CEM/AKB4) cell line by the IC_50_ of the parent (CEM) cell line.

### CEM/AKB4 cells are not cross-resistant to other classes of cytotoxic agents

To determine whether CEM/AKB4 cells are cross resistant to similar and differing classes of cytotoxic agents, cytotoxicity assays using a selective Aurora B inhibitor (AZD1152), a selective Aurora kinase A inhibitor (MLN8237), mitotic inhibitors that target tubulin (vincristine, vinblastine, paclitaxel and epothilone B), a DNA damaging agent (doxorubicin) and a multi-kinase inhibitor (ENMD2076) against CEM/AKB4 cells were compared to those for the parental CEM cell line (Table 1). CEM/AKB4 cells were 7 fold cross resistant to AZD1152 but were not resistant to any of the other drug classes. The CEM/AKB4 cells were hypersensitive to the Aurora A inhibitor MLN8237. A trend towards hypersensitivity for vincristine, paclitaxel, doxorubicin and ENMD2076 was observed but the relative resistance values were not statistically significant.

### Resistance is not due to up-regulation of multi-drug resistance proteins in CEM/AKB4 cells

ZM is thought to be a substrate of the multi-drug resistance protein P-glycoprotein and we sought to determine whether up-regulation of P-glycoprotein may mediate resistance to ZM in CEM/AKB4. Cytotoxicity assays were conducted using ZM in the presence or absence of the P-glycoprotein inhibitor verapamil. The relative resistance of CEM/AKB4 cells to ZM treated with verapamil was not significantly different to cells treated with ZM alone, showing that verapamil was not able to restore sensitivity of CEM/AKB4 to ZM and suggesting that up-regulation of P-glycoprotein is not a likely resistance pathway in these cells (Figure 1). We also excluded the role of multi-drug resistance ABCC gene family members in the resistance phenotype as there was no significant change in the expression of MDR1 or of ABCC1, 3, 4, 7, 10, or 11 in the CEM/AKB4 cells (Figure S1).

### Cell cycle analysis

**Figure 2:**
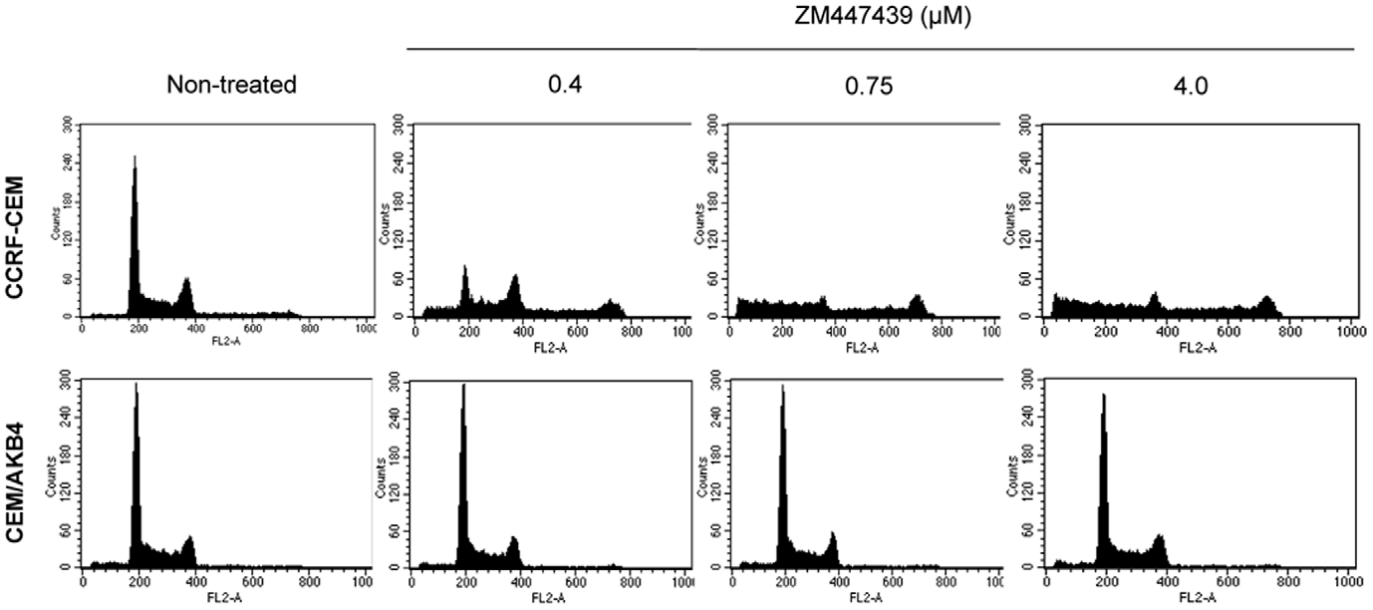
Cell cycle analysis of CEM and CEM/AKB4 cell lines. After ZM treatment (24 h), cells were harvested and assayed for their DNA content by flow cytometry. Figures are representative of three independent experiments.

Aurora B inhibitors such as ZM exert their cytotoxic effects by disrupting processes crucial for cell cycle progression. We examined the ability of ZM to induce cell cycle changes in the resistant cells using flow cytometry. Cell cycle analysis was performed on CEM or CEM/AKB4 cells treated for 24 hr in the presence or absence of 0.4, 0.75 and 4.0 µM ZM respectively (Figure 2). Without drug treatment, the cell cycle profile of CEM/AKB4 cells appeared similar to that of CEM with no observed change in proportion of cells in each phase of the cycle. Upon treatment with a low dose of ZM the profile of CEM cells showed disruption to the cell cycle consistent with inhibition of Aurora B: an increase in DNA content due to cytokinesis failure and increased sub G1 population indicative of cell death [13]. These characteristics became more pronounced with increasing drug concentration. However no changes in the profile of CEM/AKB4 cells were observed upon drug treatment even at higher concentrations (eg 4 µM) that cause massive cell death in the parental CEM cell line. Clearly the inability of ZM to exert its cell cycle disrupting effects is a pathway contributing to the resistance of the CEM/AKB4 cells.

### Aurora B is down-regulated in CEM/AKB4 cells but catalytically active

**Figure 3:**
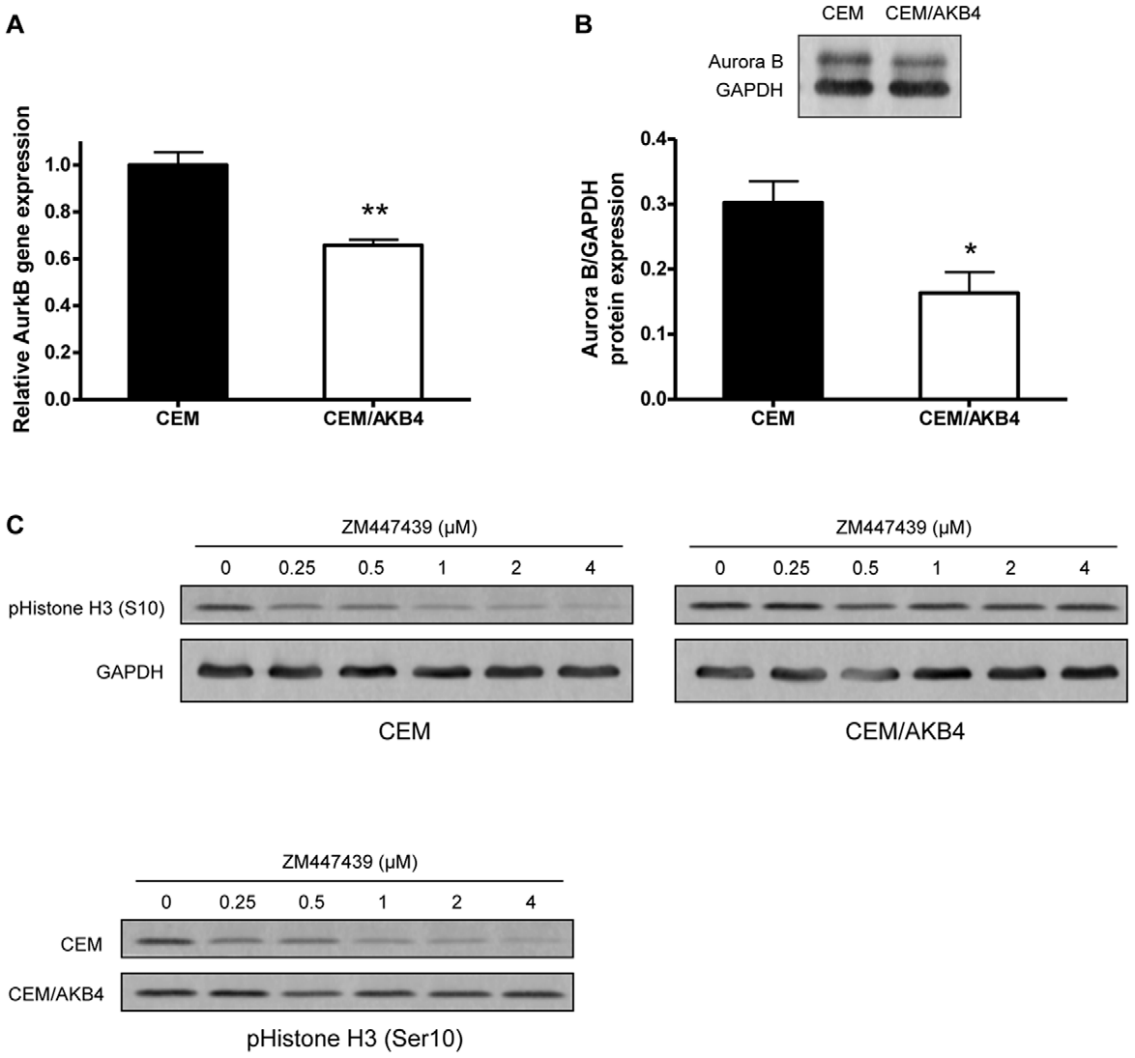
Gene and protein expression of Aurora B in CEM and CEM/AKB4 cells. (A) AurkB gene expression as determined by real-time PCR. Expression is displayed as relative ΔΔCt values of CEM/AKB4 compared to that for CEM with Ct values normalised to the cyclophilin-A gene (PPIA). (B) Aurora B protein expression determined by western blot. The densitometric volume of the Aurora B band is expressed relative to the densitometric volume of the loading control gene GAPDH. Error bars represent the SEM of three independent experiments. * p<0.05, ** p<0.005. (C) Detection of phospho Histone H3(Ser10) in CEM and CEM/AKB4 cells treated for 24 hr with increasing concentrations of ZM447439 by western blotting. Shown are representative blots from three independent experiments.

To determine whether changes in Aurora B gene and/or protein expression were associated with resistance in CEM/AKB4 cells we performed real-time PCR and western blotting. Real-time PCR analysis of cDNAs from CEM and CEM/AKB4 cells showed that gene expression of Aurora B was modestly but significantly lower in the resistant cell line (Figure 3A). Similarly, protein expression as determined by western blotting was almost 50% lower in CEM/AKB4 compared to the parental CEM cells (Figure 3B). We then asked whether catalytic activity of Aurora B is maintained in CEM/AKB4 cells in the presence of ZM447439. CEM and CEM/AKB4 cells were treated for 24 hr with increasing concentrations of ZM447439 and the levels of phosphorylated Histone H3(Ser10) determined by western blotting (Figure 3C). ZM447439 clearly suppressed H3(Ser10) phosphorylation in the parental CEM cells, however, levels of phosphorylated H3(Ser10) were relatively unchanged in CEM/AKB4 cells when treated with up to 4 µM ZM447439. Additionally we performed similar gene and protein expression analyses for Aurora A to determine whether resistance may be mediated through an Aurora A dependent pathway. No differences in either gene or protein expression of Aurora A in CEM and CEM/AKB4 cells were observed (data not shown).

To address whether the localization of Aurora B was altered in the resistant CEM cells, immunofluorescence staining was employed. As expected, in CEM cells Aurora B is maximally expressed in mitotic cells and localises to centromeres in metaphase, to the spindle midzone in anaphase/telophase and to the midbody in cytokinesis (Figure S2). In several independent experiments no difference in Aurora B localization was observed between CEM and CEM/AKB4 cells. The mitotic indices for both CEM and CEM/AKB4 cells were obtained in the presence and absence of 4 µM ZM447439 and no significant differences were observed either in basal levels or drug treated levels (Table 2). Collectively, these results suggest that despite decreased expression levels, localization and catalytic function of Aurora B is not impaired in resistant CEM/AKB4 cells compared to CEM.

**Table 2:** Mitotic index of CEM and CEM/AKB4 cells in the presence and absence of 4 µM ZM447439.

	Mitotic index (%)*	
Cell line	Untreated	4 µM ZM447439
CEM	7.80±0.56	8.89±0.46
CEM/AKB4	8.27±0.28	8.32±0.61

*Percentages of mitotic cells were calculated after counting at least 1000 cells in three independent experiments.

### CEM/AKB4 cells express a point mutation in Aurora B

Point mutations in the catalytic domain are known to confer resistance of cancer cells to kinase inhibitors so we sought to determine whether kinase domain or other mutations are contributing to the resistant phenotype in CEM/AKB4 cells. Accordingly the full length sequence of the Aurora B gene was obtained and compared between CEM and CEM/AKB4 cells. As ZM447439 is known to inhibit Aurora A the full length sequence of this gene was also determined. The resistant CEM/AKB4 cells featured a single point mutation in the kinase domain of Aurora B that gives rise to a G160E amino acid substitution (Figure S2). This residue lies in the hinge region of the catalytic domain of the protein, an important site involved in Aurora B inhibitor binding [34]. In contrast, no mutations were detected in the Aurora A gene were detected.

### G160E substitution impairs Aurora B inhibitor binding

**Figure 4:**
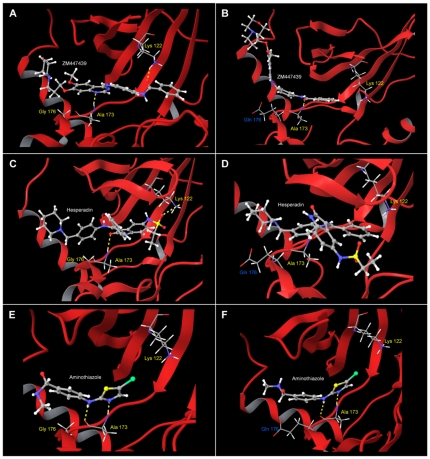
Docking of Aurora B inhibitors with the catalytic domain of wild-type and mutant Aurora B with the G160E substitution (G176E for xenopus laevis). Docked poses were compared between wild-type and mutant Aurora B for (A, B) ZM447439, (C, D) hesperadin and (E, F) aminothiazole inhibitor. Hydrogen bonds referred to in the text highlighted in yellow.

Interestingly the G160E substitution has also been described in ZM resistant colorectal cancer cells suggesting that this is an important residue in ZM binding [21]. The mutation has been presumed to mediate resistance by hindrance of drug binding through steric interactions with the bulkier glutamate residue. To further elucidate the role of the G160E mutation we used a molecular modelling approach with docking studies to explore the influence of this substitution on Aurora B inhibitor binding and resistance mechanisms. In our methodology the initial templates were based upon available crystal structures of inhibitors bound to xenopus laevis Aurora B from whence we employed docking calculations with the corresponding inhibitor as described in the Materials and Methods section. The three inhibitors and their corresponding crystal structure PDB entries were ZM447439 (2VRX), hesperadin (2BFY) and an aminothiazole inhibitor (2VGP) with the starting templates prepared by removing the drug molecule from the crystal structure and substituting glycine at the 160 position (176 in xenopus laevis Aurora B) for glutamate for the case of the mutant docking calculations. Each drug was then docked into the ATP binding pocket with calculations yielding several docked poses. Examination of the docked poses in wild type Aurora B showed that the drug molecules adopted similar conformations and binding modes to those observed in the corresponding crystal structures, validating the models and our methodology (Figure S3). These calculations showed that ZM and hesperadin formed hydrogen bonds to the Ala173 (2.602 Å for ZM, 2.897 Å for hesperadin) and Lys122 (1.889 Å for ZM, 2.873 Å for hesperadin) residues of Aurora B that have been previously demonstrated to be key interactions for potent Aurora B inhibition [35], [36]. The aminothiazole inhibitor on the other hand formed hydrogen bonds to Ala173 (2.00 Å) and Leu99 (2.974 Å) but not Lys122 and this alternative binding motif has been postulated to be responsible for a different mode of action for this drug. ATP was also docked into the binding cavity and likewise assumed similar poses to confirmations observed in crystal structure determinations (Figure S4A). We then repeated the same docking calculations in the mutant Aurora B templates. Initially ATP was docked into the mutant enzyme and importantly showed similar binding patterns and orientations as observed in the wild-type enzyme (Figure S4B), suggesting that catalytic activity of Aurora B is maintained in the presence of the mutation. Docking of the ZM and hesperadin molecules into the mutant Aurora B containing the bulkier Gln176 residue produced poses significantly different to those in the wild type enzyme (Figure 4). These inhibitors did not penetrate as deep into the binding pocket as for the wild type enzyme even though this cavity in the mutant is still relatively large. In particular the ZM molecule resides largely outside this region. Moreover both molecules adopted different orientations in the binding site of the mutant compared to wild-type enzymes introducing alternative chemical moieties into this region. The active binding motif present in docking in the wild type Aurora B was missing, with hydrogen bonds to Lys122 absent for both molecules. According to our criteria, therefore, none of the docked poses corresponded to a conformation that would significantly inhibit kinase activity of Aurora B. In contrast, the docked poses adopted by the aminothiazole inhibitor in the mutant Aurora B were nearly identical to those observed in the wild type with the same orientation and hydrogen bonding patterns present.

### Increased ZM447439 selective pressure leads to increased gene expression of MDR1

**Figure 5:**
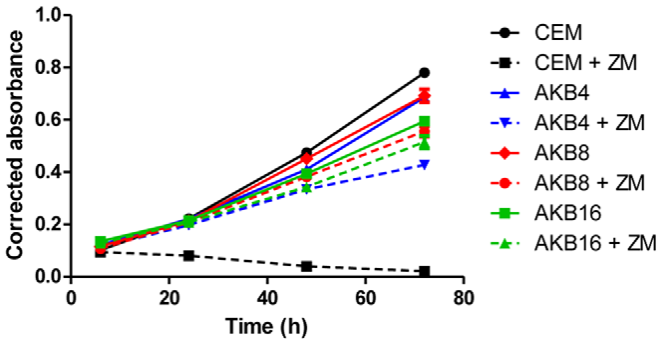
Proliferation timecourse of CEM and CEM/AKB cells in the presence and absence of ZM447439. Cells were grown either in vehicle alone or in 4 µM ZM447439 and proliferation was determined at indicated timepoints as the corrected absorbance using the Alamar blue assay measured spectrophotometrically. Error bars represent the SEM of three independent experiments.

**Figure 6:**
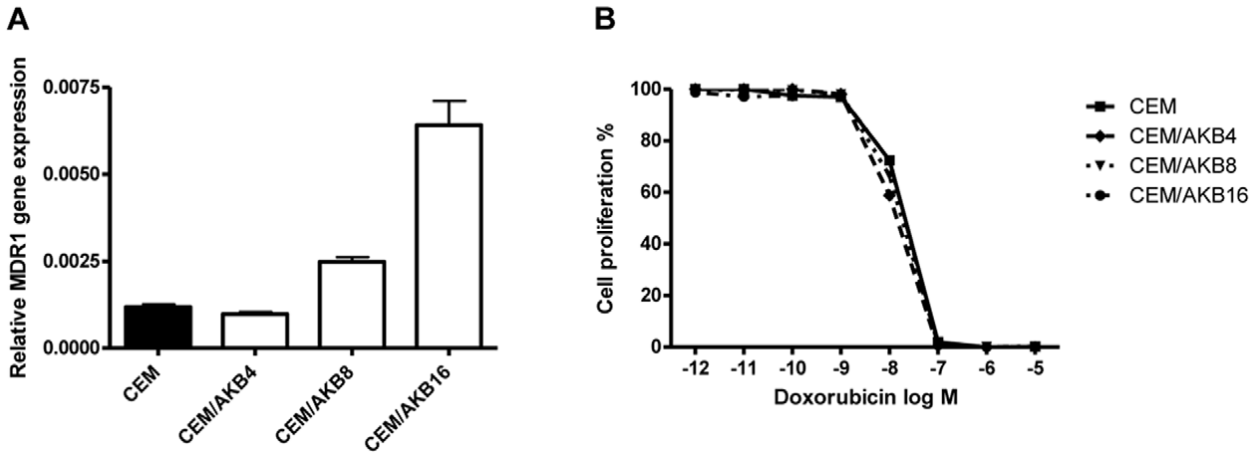
Expression and functional relevance of MDR1 (P-glycoprotein) in CEM/AKB cells. A) CEM/AKBMDR1 mRNA expression in ZM447439 resistant CEM cells as determined by real-time PCR. Expression is displayed as relative ΔΔCt values of CEM, CEM/AKB4, CEM/AKB4 and CEM/AKB4 cells compared to that for CEM/VCRR cells with Ct values normalised to the cyclophilin-A gene (PPIA). Error bars represent the SEM of three independent experiments. B) Cytotoxicity assays of doxorubicin against CEM and CEM/AKB4, 8, and 16 cells.

A key question was whether increasing drug selective pressure on the CEM/AKB4 cells would lead to more highly resistant cells with additional resistance mechanisms. To address this we examined resistance mechanisms in cells more highly resistant to ZM447439, with the CEM/AKB8 and CEM/AKB16 sublines generated by sequential treatments of CEM/AKB4 cells with 8 µM and 16 µM ZM447439 respectively. The CEM/AKB8 and CEM/AKB16 sublines were 14.8- and 155-fold resistant to ZM447439 respectively compared to parental CEM cells as determined by cytotoxicity assays. Proliferation of the cells compared to CEM cells was determined in the presence and absence of 4 µM ZM447439 and showed that basal levels of proliferation across all cells were not appreciably different (Figure 5). As observed for CEM/AKB4 cells, CEM/AKB8 and CEM/AKB16 cells continued to proliferate in the presence of the selecting agent (Figure 5). Gene and protein expression of Aurora B was analysed to establish whether any alterations may be mediating the increased resistance of CEM/AKB8 and CEM/AKB16 cells. Interestingly, while both gene and protein expression of Aurora B in CEM/AKB4 cells were lower than CEM cells, expression levels reverted to near equivalence with increasing selective pressure (Figure S5). Full length sequencing of the Aurora B gene in CEM/AKB8 and CEM/AKB16 cells showed the G160E substitution present in CEM/AKB4 cells was preserved, however no additional point mutations were found. Gene and protein expression of Aurora A was analysed but no differences were identified between CEM cells and CEM/AKB8 and CEM/AKB16 cells (data not shown). Moreover, no mutations in Aurora A were detected. To establish whether up-regulation of multidrug resistance proteins was associated with a higher level of resistance to ZM447439, the expression of MDR1 and ABCC1, 2, 3 and 4 genes in CEM/AKB8 and CEM/AKB16 cells was determined by real-time PCR. Whilst expression of MDR1 mRNA was not appreciably altered in CEM/AKB4 cells compared to CEM, levels increased in a dose dependant manner for CEM/AKB8 and CEM/AKB16 cells, with approximately 2- and 5-fold increases respectively (Figure 6A). However the increased MDR1 expression was not functionally relevant as sensitivity to doxorubicin, a P-glycoprotein substrate, was not altered in CEM/AKB16 cells compared to CEM cells using cytotoxicity assays (Figure 6B). Uptake of Daunorubicin, another P-glycoprotein substrate, was not reduced in these same cells as determined by flow cytometry (data not shown). Expression of ABCC1, 2, 3 and 4 was unaltered in all CEM/AKB cells compared to CEM cells (data not shown).

### CEM/AKB16 cells are resistant to apoptosis and Aurora B inhibition

**Figure 7:**
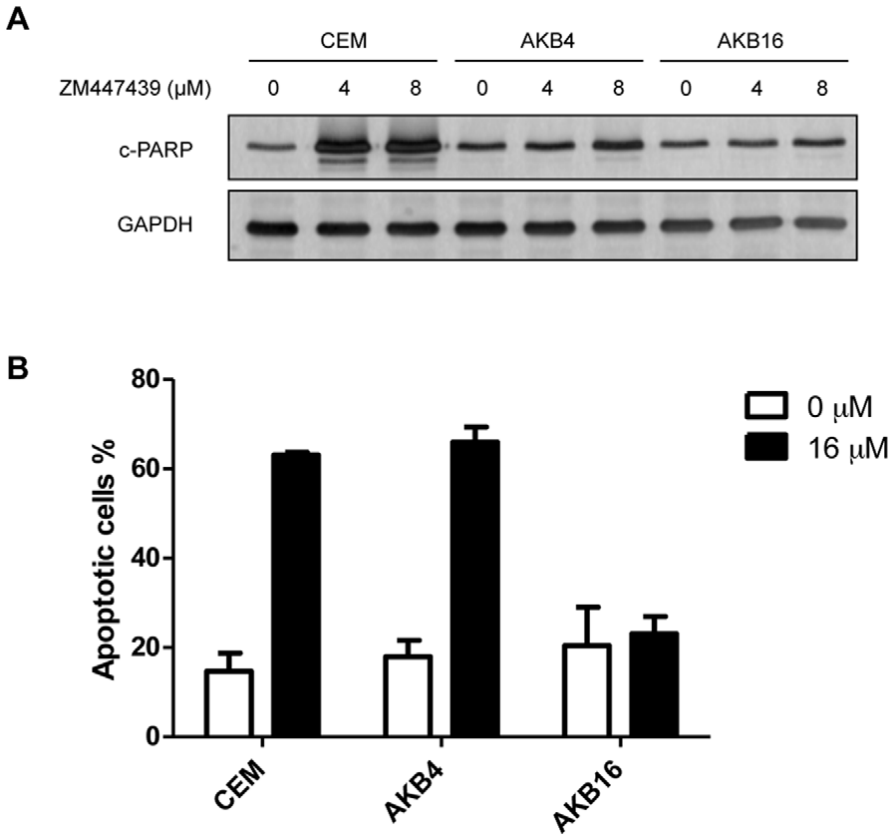
Induction of apoptosis in CEM and CEM/AKB cells. A) Levels of cleaved PARP in cells treated with indicated concentrations of ZM447439 for 24 h as determined by western blot. B) Proportion of apoptotic cells in both untreated and CEM and CEM/AKB cells treated with 16 µM ZM447439 for 24 h.

Given that the CEM/AKB16 cells are highly resistant to ZM447439 and this is not due to additional mutations in Aurora kinase B, or reduced drug transport, we focused on the ability of the CEM/AKB16 cells to undergo apoptosis in the presence of drug. CEM/AKB16 and CEM cells were treated with increasing concentrations of drug and monitored for the expression of markers of apoptosis after 24 hr (Figure 7). Apoptosis indicated by cleavage of PARP, a substrate of the apoptotic caspases, is strongly induced in CEM cells by treatment with 4 and 8 µM ZM447439, however the level of this induction is far less in CEM/AKB4 and CEM/AKB16 cells (Figure 7A). Furthermore, upon treatment with 16 µM ZM447439 for 24 hr the proportion of apoptotic cells as determined by Annexin V-FITC staining is increased for CEM and CEM/AKB4 cells compared to control untreated cells, yet remains unchanged in CEM/AKB16 cells (Figure 7B). Together these results suggest that resistance to apoptosis is a primary mechanism mediating the phenotype of CEM/AKB4 and also the more highly resistant CEM/AKB16 cells.

**Figure 8:**
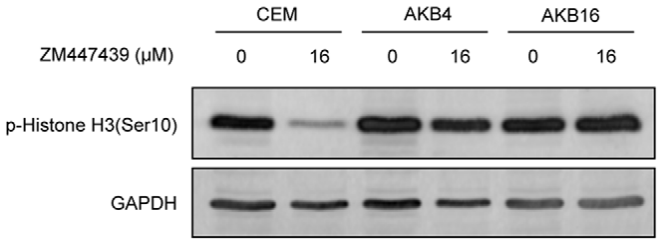
Levels of phosphorylated Histone H3 in CEM and CEM/AKB cells in the presence or absence of 16 µM ZM447439.

To determine whether the high level resistance of CEM/AKB16 to ZM447439 is mediated by inhibition of Aurora B, or another pathway, the levels of phosphorylated Histone H3(ser10) in cells treated with 16 µM ZM were analysed by western blotting (Figure 8). As expected, CEM cells treated with 16 µM ZM have dramatically lower levels of phospho H3 compared to untreated cells consistent with inhibition of Aurora B. However phospho H3 levels in both treated and untreated CEM/AKB4 and CEM/AKB16 cells are not appreciably different. This data strongly suggests that Aurora B remains catalytically active in the presence of high drug concentrations and this may be mediating the highly resistant phenotype in the CEM/AKB16 cells.

## Discussion

Understanding the molecular factors that contribute to sensitivity and resistance to new chemotherapeutic agents is crucial to their effective implementation in treatment regimes. Moreover, establishing the drug-target interactions mediating these processes allows for the rational design of more potent and effective molecules. Herein we have described the development and characterisation of Aurora B inhibitor resistant leukemia cell lines that have acquired multiple genetic defects including i) a point mutation in the Aurora B kinase domain and ii) decreased ability to undergo apoptosis. Hematological malignancies have proven to be particularly responsive to these agents in early clinical evaluation and hence our findings could be important to optimise future efficacy against leukemia.

Characterisation of CEM/AKB4 cells revealed that resistance is not mediated by multidrug resistance pathways. CEM/AKB4 cells were not cross-resistant to a broad range of cytotoxic agents, including an Aurora A inhibitor, and moreover, did not show transcriptional activation of ABCC family drug transporters. The CEM/AKB4 cells were hypersensitive to the Aurora A inhibitor MLN8237. CEM/AKB4 cells were, however, cross resistant to a selective Aurora B inhibitor, AZD1152, indicating an Aurora B dependant mechanism of resistance. Although ZM447439 is known to inhibit Aurora A we excluded the possibility of an Aurora A dependent mechanism contributing to resistance to these cells by the lack of Aurora A gene and protein alterations in CEM/AKB4 cells and a lack of cross resistance to the selective Aurora A inhibitor MLN8237. This is in agreement with other reports that show the cytotoxic activity of ZM447439 is mediated through Aurora B, not Aurora A inhibition [16]. Detection of a G160E point mutation in the kinase domain of Aurora B suggested that resistance in CEM/AKB4 cells is mediated through impaired binding of the drug to the target kinase. Genetic alterations to drug targets are common mechanisms mediating resistance to targeted therapies; point mutations in BCR-ABL conferring resistance to Imatinib in leukaemia is a classic example. Moreover, the G160E mutation in Aurora B has been reported in colorectal cells selected for resistance to ZM447439. Our findings in a leukaemia cell line further validate that the 160 position is particularly important for drug binding and that point mutations of this residue afford highly penetrant resistance. This mutation should be validated in a clinical setting as it may be important in the use of Aurora B inhibitors and resistance to therapy, much as the T315I BCR-ABL mutation is highly prognostic of outcome for Imatinib treatment in CML patients. As yet, the G160E mutation has not been reported in studies of Aurora B inhibitors in animal models or clinical studies.

Although the Aurora B G160E substitution has been shown to independently confer resistance to Aurora B inhibitors it has not been conclusively shown how drug binding is affected. We therefore employed a molecular modelling approach to understand how the G160E substitution alters drug binding and to gain further insights into drug-target interactions of Aurora B inhibitors. Our docking results confirm that binding of ATP to Aurora B is unaltered in mutant Aurora B compared to the wild-type, thereby maintaining catalytic activity. We showed that hydrogen bonding of Aurora B inhibitors to the Ala173 and Lys122 residues are key interactions mediating drug activity by preventing catalytic binding of ATP. However, the presence of the G160E mutant hinders the ability of inhibitors to penetrate as far into the binding pocket as the wild-type enzyme precluding the formation of these hydrogen bonds. Presumably inhibitors are only able to bind to the mutant enzyme in modes that do not compete effectively with ATP and substrate binding, thereby allowing catalytic activity in the presence of the drug and a resistant phenotype. It would be expected that any Aurora B inhibitor that has a similar active binding motif would be affected, explaining the cross resistance of cells with this mutation to structurally related inhibitors in our studies and others [21]. Our models could therefore be used as a screen to identify, or rationally design, inhibitors with novel binding modes that may abrogate Aurora B G160E mediated resistance.

The progression of resistance with repeated or higher concentration drug exposure is an important consideration in the treatment of relapsed disease. Both CEM/AKB8 and CEM/AKB16 cells showed a dose dependent increase in transcriptional activity of MDR1, however P-glycoprotein was not functionally active in either case. Moreover, both parental CEM cells and resistant CEM/AKB8 and CEM/AKB16 cells were equally sensitive to doxorubicin suggesting an absence of a multidrug resistance phenotype. Nevertheless, CEM/AKB16 cells showed an increased resistance to apoptosis as measured by levels of c-PARP and Annexin V. Resistance to kinase inhibitors may also be effected by aberrant activation of redundant signalling pathways to that of the target, an example being MET amplification in resistance to EGFR kinase inhibitors [37]. As CEM/AKB16 cells were highly resistant to Aurora B inhibition it appears that sustained Aurora B activity in the presence of ZM447439 may still be driving resistance in these cells rather than activation of an alternative pathway. Previous work from our laboratory on drug resistance mediated by tubulin mutations showed that CEM cells acquire additional point mutations in tubulin at higher levels of resistance [26]. Both CEM/AKB8 and CEM/AKB16 cells expressed the Aurora B G160E mutation described for CEM/AKB4 cells, however no additional mutations in Aurora B were observed, further demonstrating the importance of the 160 residue in drug binding and high-level resistance.

Our study of phosphorylated Histone H3 levels showed that CEM/AKB4 cells maintain resistance to Aurora B inhibition at 16 µM ZM, despite this drug concentration being sufficient to induce apoptosis and cell death. This is consistent with off-target kinase inhibition of ZM447439, where at high drug concentrations the contribution of targeting additional cytotoxic pathways to Aurora B inhibition becomes significant. Therefore the resistant phenotype in CEM/AKB16 cells may potentially be mediated through alterations in these other targets of ZM447439. ZM447439 has been shown to potently inhibit Aurora A as well as Aurora B in biochemical assays [18] and we analysed CEM/AKB16 cells for alterations in Aurora A. We found no changes in gene or protein expression of Aurora A in CEM/AKB16 cells and no mutations in the Aurora A gene (data not shown). Additionally, CEM/AKB16 cells were as equally sensitive as CEM cells to a selective Aurora A inhibitor MLN8237 (data not shown), suggesting that ZM447439 resistance in these cells is not mediated through an Aurora A dependent pathway. It is possible that alterations in other unknown targets of ZM447439 may be responsible, and ultimately, an understanding of the precise mechanisms underpinning resistance in the more highly resistant CEM/AKB8 and CEM/AKB16 cells will shed further light on the mode of action of this drug.

Aurora B inhibitors remain a promising area for targeted anticancer therapy, yet a fuller understanding of drug response and resistance mechanisms will aid their clinical implementation. Our findings have confirmed that resistance to these agents is likely across a variety of malignancies and that point mutations in Aurora B, particularly of the 160 residue, may be highly significant markers of treatment outcome. Moreover, our analysis of highly resistant cells suggests that sustained or high-level drug treatment may give rise to an evolution of multiple mechanisms of resistance in patients. Accordingly, our models provide a basis for designing and testing alternative Aurora B inhibitors, and for screening agents that may be employed in combination therapeutic approaches.

## Supporting Information

Figure S1 **Relative gene expression of common ABCC drug transporter proteins in CEM/AKB4 cells compared to parental CEM cells.** Expression was determined by real-time PCR using Taqman probes for MDR1 and ABCC1-12. Ct values were normalised to PPIA and expression calculated by the ΔΔCt method. No expression was observed for ABCC3, 5, 6, 8, 9, 12 in either CEM or CEM/AKB4.
(PDF)

Figure S2 **Localisation of Aurora B in mitotic CCRF-CEM cells compared with CEM/AKB4 cells by immunofluorescence staining.** Cells were stained for Aurora B, α-tubulin, and DNA (DAPI). Scale bar = 10 µM.
(PDF)

Figure S3 **Comparison between crystal structure of Aurora B inhibitors cocrystallised with Aurora B and docking of corresponding inhibitor with Aurora B used to validate the methodology.**
(PDF)

Figure S4 **Docking of ATP with the catalytic domain of wild-type and mutant Aurora B with the G160E substitution (G176E for xenopus laevis).** Docked poses were compared between (A) wild-type and (B) mutant Aurora B.
(PDF)

Figure S5 **Gene and protein expression of Aurora B in CEM and CEM/AKB cells.** (A) AurkB gene expression as determined by real-time PCR. Expression is displayed as relative ΔΔCt values of CEM/AKB4, AKB8 and AKB16 cells compared to that for CEM with Ct values normalised to the cyclophilin-A gene (PPIA). (B) Aurora B protein expression determined by western blot. The densitometric volume of the Aurora B band is expressed relative to the densitometric volume of the loading control gene GAPDH. Error bars represent the SEM of three independent experiments.
(PDF)
