# Supplementary figures and images for: Evolution of Resistance to Aurora Kinase B Inhibitors in Leukaemia Cells
Source: PLoS One. 2012 Feb 16;7(2):e30734. doi: 10.1371/journal.pone.0030734 (PMC3281142; doi:10.1371/journal.pone.0030734)

## Supplementary Information

Figure S1

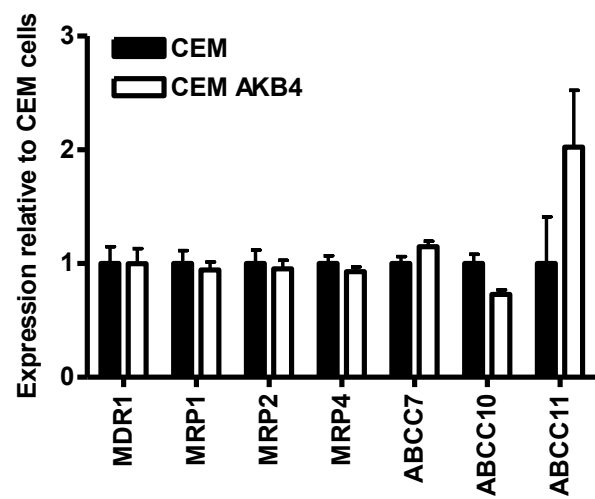

Supplement: Figure S1 — Relative gene expression of common ABCC drug transporter proteins in CEM/AKB4 cells compared to parental CEM cells. Expression was determined by real-time PCR using Taqman probes for MDR1 and ABCC1-12. Ct values were normalised to PPIA and expression calculated by the ΔΔCt method. No expression was observed for ABCC3, 5, 6, 8, 9, 12 in either CEM or CEM/AKB4. (PDF) [file pone.0030734.s001.pdf]

Figure S2.

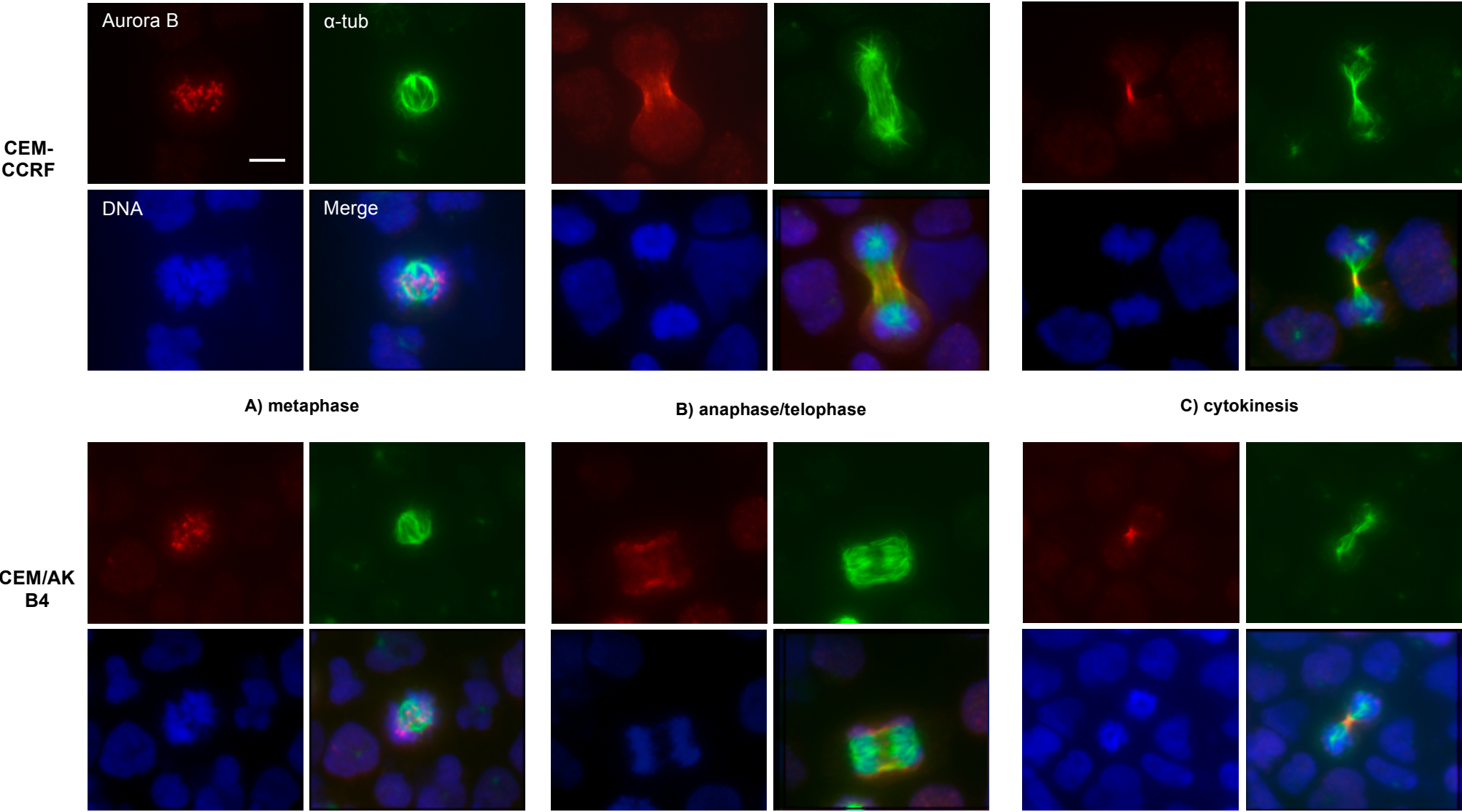

Supplement: Figure S2 — Localisation of Aurora B in mitotic CCRF-CEM cells compared with CEM/AKB4 cells by immunofluorescence staining. Cells were stained for Aurora B, α-tubulin, and DNA (DAPI). Scale bar = 10 µM. (PDF) [file pone.0030734.s002.pdf]

Figure S3.

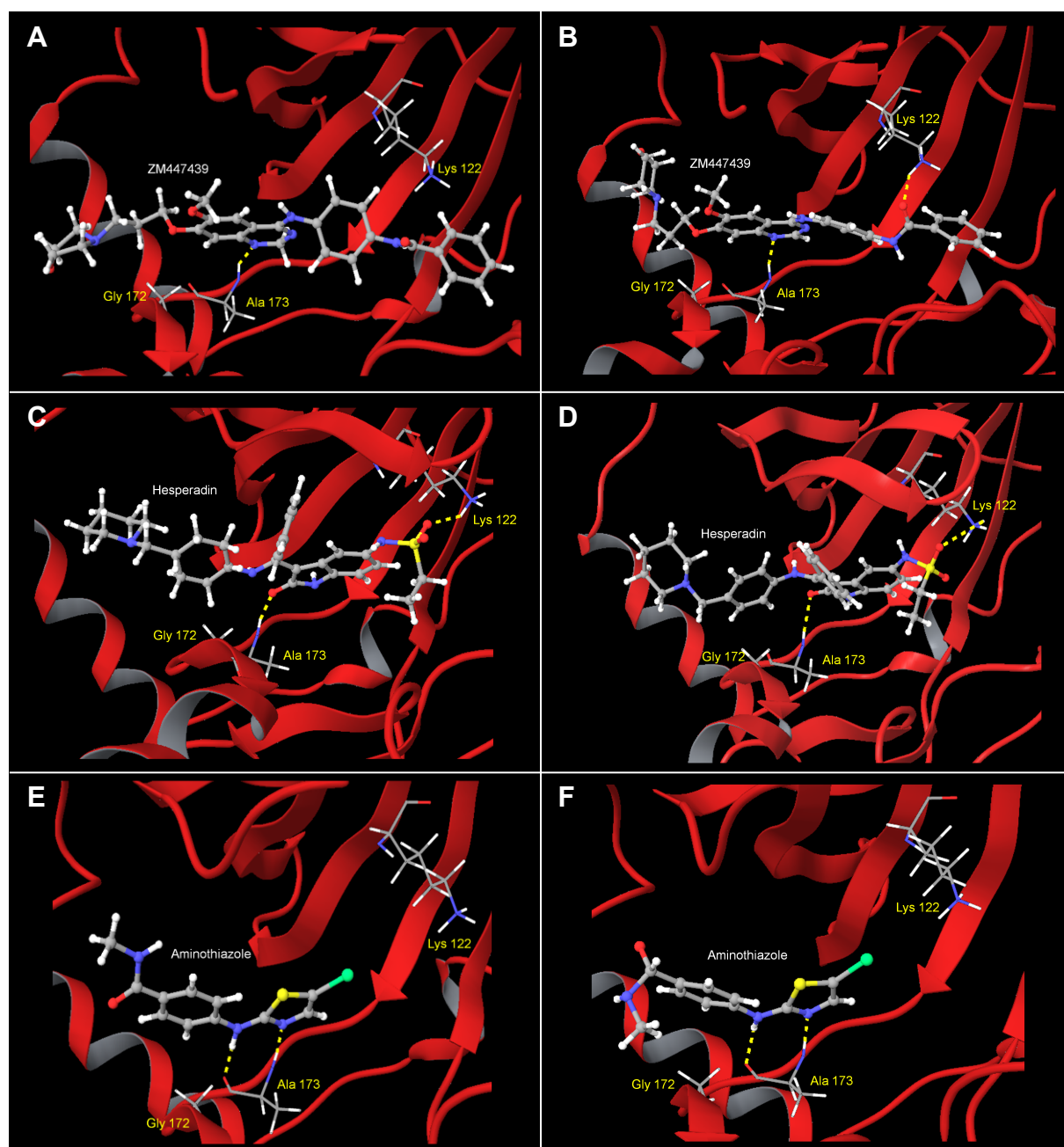

Supplement: Figure S3 — Comparison between crystal structure of Aurora B inhibitors cocrystallised with Aurora B and docking of corresponding inhibitor with Aurora B used to validate the methodology. (PDF) [file pone.0030734.s003.pdf]

**Figure S4.**

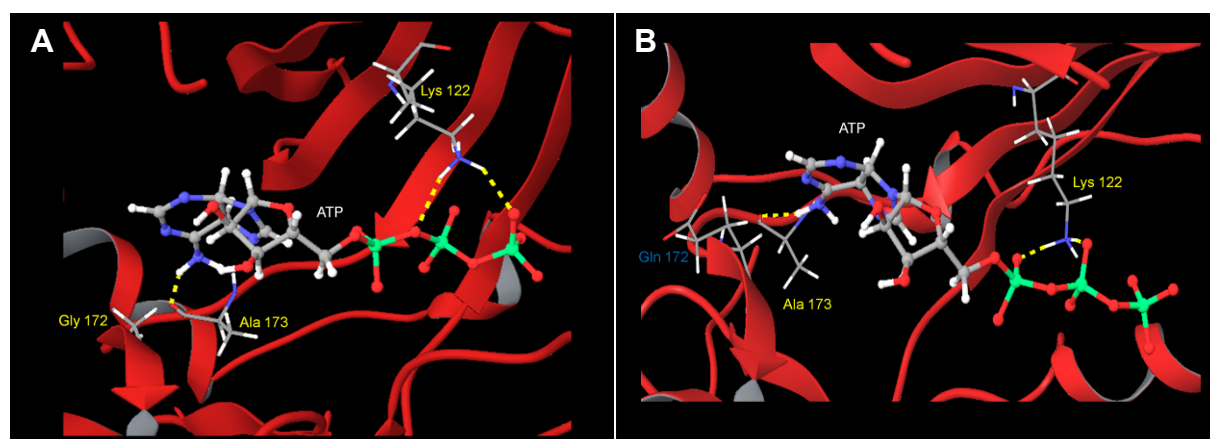

Supplement: Figure S4 — Docking of ATP with the catalytic domain of wild-type and mutant Aurora B with the G160E substitution (G176E for xenopus laevis). Docked poses were compared between (A) wild-type and (B) mutant Aurora B. (PDF) [file pone.0030734.s004.pdf]

Figure S5.

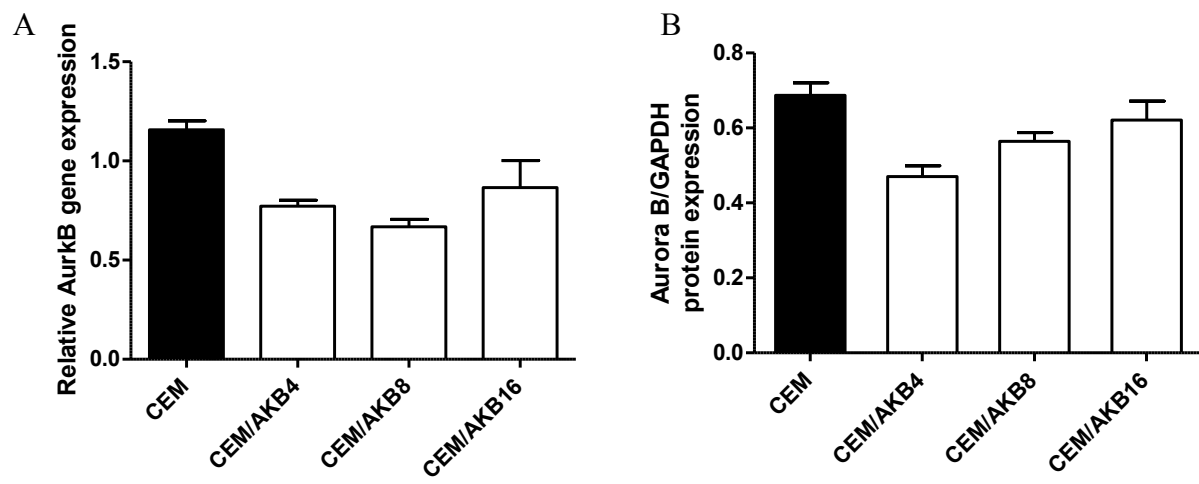

Supplement: Figure S5 — Gene and protein expression of Aurora B in CEM and CEM/AKB cells. (A) AurkB gene expression as determined by real-time PCR. Expression is displayed as relative ΔΔCt values of CEM/AKB4, AKB8 and AKB16 cells compared to that for CEM with Ct values normalised to the cyclophilin-A gene (PPIA). (B) Aurora B protein expression determined by western blot. The densitometric volume of the Aurora B band is expressed relative to the densitometric volume of the loading control gene GAPDH. Error bars represent the SEM of three independent experiments. (PDF) [file pone.0030734.s005.pdf]
